# Investigating the effects of bus numbering in a radial transmission network using load-flow study

**DOI:** 10.1016/j.heliyon.2021.e07098

**Published:** 2021-05-27

**Authors:** Ademola Abdulkareem, A.S. Alayande, T.E. Somefun, E.V. Ette

**Affiliations:** a Electrical and Information Engineering Department, College of Engineering, Covenant University, Ota, Nigeria; b Department of Electrical and Electronics Engineering, Faculty of Engineering, University of Lagos, Nigeria

**Keywords:** Bus identification number, Convergent failure and line losses, MATLAB, Newton-Raphson method, Power World Simulator

## Abstract

This paper explains the effects of bus numbering using a load-flow study to investigate the Nigerian 330 kV radial transmission networks. The objectives of this research include the Newton-Raphson-based load-flow analysis and verification of power losses. The simulation of the load-flow analysis is carried out using the software of Power World Simulator and MATLAB, while verification of power losses is simulated with only MATLAB software. The Nigerian 330-kV transmission lines used in this study are radial and are overloaded; thus, it has been subjected to numerous studies covering many areas as to how improvements can be made. All these studies aimed at increasing the efficiency of the network and reduce real and reactive power losses. In this study, analysis is carried out on the failure to convergence; the result of load-flow as obtained for the 28-bus power system of the Nigerian 330-kV network using two different bus identification numbering sequence types. The results of the Newton-Raphson load-flow solution in Power World Simulator and MATLAB platform obtained for each of the two bus identification types revealed the convergence failure in one identification model numbering type. This result's inconsistency further necessitated the study of load-flow analysis on the Nigerian 330-kV network for other different bus identification numbering types as reviewed from past work for case studies. The same bus data and transmission line data obtained from PHCN are used for all the bus numbering model types generated in the study. The results revealed variations in the real and reactive power losses and the number of iterations in solving each case. Besides, the study discovered that the failure in convergence comes from the power solution method's failure (software) used, hence, a code-based platform should be used for verification.

## 1 Introduction

The demand for electrical energy has always been on the increase simply because of population growth and industrial development in developing countries [1]. Hence, the operation of transmission lines at maximum efficiency is very important [2, 3]. Power system quality and its analysis are irreplaceable when it comes to the generation, transmission, and distribution of electrical power either in small or large-scale power system networks. However, today's power system is a complex network of transmission lines interconnecting the generating units of various locations to the major load points. The load-flow analysis on power systems provides various iterative techniques of solution that determine the steady-state of line flow and losses, line currents, and line voltages for the best operation and planning of power system [4]. This analysis cannot be overemphasized in a country like Nigeria where the generation, transmission, and distribution of power have been a major setback in the nation's development.

The literature on load-flow solutions (Newton-Raphson or Gauss-Seidel) analysis performed on Nigeria 330kV network is almost in flux, but a regular problem facing researchers for so long is finding the solution to the inaccuracy in the correct assessment of the flow of active and reactive power and line losses [5, 6]. In most cases, the magnitude, as well as the location of the voltage, violated buses are fairly determined, but there are discrepancies in the values of active and reactive powers and the overall losses load flow analysis. Ignatius did a load-flow study on the Nigerian 330-kV power system and discovered that there are more than a few losses on [7]. When a load flow study is carried out, the core of the study is to find out the complex bus voltages, active and reactive power losses. The first data required for load-flow studies is the bus identification data; numbering sequentially, all the nodes of the system from 1 to n. It includes numbers of PV buses, load buses, and line buses [8]. However, it has been observed from the literature review of load-flow analysis performed on the Nigerian 330-kV network that the sequence of bus identification numbering used by various researchers is not the same in most cases. This different bus numbering results in inconsistent real and reactive power losses and inaccurate voltage violation magnitude.

Similar research related to load flow studies, for instance, Manirakiza and Ekwue carried out load-flow using bus identification data such as bus 1 for Oshogbo, bus 2 Benin, bus 3 Ikeja, etc. in the estimation of technical losses in the Nigerian 330-kV transmission network [9]. Abdulkareem performed load-flow to investigate the real and reactive power losses and voltage violation buses in the existing Nigerian 330-kV transmission network using two generated bus identification numbering models: one model with Egbin (#1), Afam (#2), Ayede (#3), etc. and the second model, Kanji (#1), Birnin-Kebbi (#2), Jebba T.S (#3), etc. [10] Samuel et al. carried-out load flow studies in the Nigerian 330-kV network to investigate the selection of a suitable slack bus in multi-generation stations using bus identification numbering such as Egbin (#1), Delta (#2), Aja (#3), etc. and observed from the generated results that a change in the slack bus active power, reactive power and losses on the line due to the influence of phase angle [11]. Adepoju et al. on the other hand, introduced FACTs Controllers on the Nigerian transmission network using different bus identification numbering type [12]. Adedapo and Izuegbunam et al. also carried out contingency assessment of the Nigerian 330-kV power grid using a completely different bus identification numbering data [13, 14]. Maruf and Garba, Onojo et al., and Ogbuefi and Madueme have to determine bus voltage and power losses and analyze load-flow of the Nigerian 330-kV grid using different bus identification numbering types [15, 16, 17]. Also, Adebayo et al. performed the steady-state voltage stability enhancement of the Nigerian 330-kV grid system with a different bus numbering type [18].

It is evident from the above review that so much has been done on the analysis of the power system to losses and voltage violations at the buses, but most of the time the effect of the numbering system on the convergence of the system is neglected. However, bus numbering has been systematically examined using the load-flow study to influence the real and reactive powers magnitude, the line losses, and voltage violation buses as unveiled in this study. This study aims to determine the bus identification numbering system's effect on load-flow analysis by generating random numbering systems using Power World Simulator and MATLAB software. The randomly generated numbering is then used to analyze the existing Nigerian 330-kV transmission network. The results are compared for the different numbering systems to determine their effect on the system's losses and convergence using MATLAB.

## 2 Methodology

The data collected from PHCN includes Line, bus, data and generator data. Several different bus identification numbering types were generated for the Nigerian 330-kV, 28-bus network and the single line diagram was redrawn for the identified 8-buses for ease of load flow analysis using the Power-World and MATLAB environments. The effects of the bus identification numbering types on the active and reactive power losses are then considered for each case. The implementation of this simulation is related to Newton-Raphson method, Power World Simulator, and generated bus identification numbering types.

### 2.1 Newton Raphson method

The method uses the iterative approximation technique of Newton-Raphson algorithm to solve the nonlinear problem with the help of an initial guess for unspecified parameters and Taylor's series approach for expansion is shown on Eq. (1).

(1) $${\text{Ι}}_{i}=V_{i}\overset{n}{\underset{j=0}{\sum }}y_{ij}-\overset{n}{\underset{j=1}{\sum }}y_{ij}V_{j\;j\neq i}.$$

In terms of bus admittance matrix, Eq. (1) becomes to Eq. (2).

(2) $${\text{Ι}}_{i}=\overset{n}{\underset{j=1}{\sum }}Y_{ij}V_{j\;j\neq i}$$

Expressing the above equation in polar form results is shown in Eq. (3)

(3) $${\text{I}}_{\text{i}}\;=\overset{n}{\underset{j=1}{\sum }}\left|Y_{ij}\right|\left|V_{j}\right|∠\theta_{ij}-\delta_{j}$$

Complex power is obtained from

(4) $$P_{i}-jQ_{i}=V_{i}^{*}I_{i}.$$

(5) $$P_{i}-jQ_{i}=\left|V_{i}\right|∠-\delta_{i}\overset{n}{\underset{j=1}{\sum }}\left|Y_{ij}\right|\left|V_{j}\right|∠\theta_{ij}+\delta_{j}$$

Based on Eq. (5), two equations can be extracted which result in Eq. (6) and Eq. (7), respectively.

(6) $$P_{i}=\overset{n}{\underset{j=1}{\sum }}\left|V_{i}\right|\left|V_{j}\right|\left|Y_{ij}\right|\text{cos}\left(\theta_{ij}-\delta_{i}+\delta_{j}\right)$$

(7) $$Q_{i\;=}-\overset{n}{\underset{j=1}{\sum }}\left|V_{i}\right|\left|V_{j}\right|\left|Y_{ij}\right|\text{sin}\left(\theta_{ij}-\delta_{i}+\delta_{j}\right)$$

Newton Raphson method given in terms of the Jacobian matrix is shown in Eq. (8).

(8) $$\left[\begin{array}{l} \text{Δ}P\\ \text{Δ}Q \end{array}\right]=\left[\begin{array}{ll} J_{1} & J_{2}\\ J_{3} & J_{4} \end{array}\right]\left[\begin{array}{l} \text{Δ}\delta \\ \text{Δ}\left|V\right| \end{array}\right]$$

where;

ΔP and  ΔQ are the active and reactive mismatch of power

Δ|V| and  Δδ are bus voltage magnitude and angle

J_1_, J_2_, J_3_, and J_4_ are known as Jacobian sub-matrices

The Jacobian submatrices can be obtained from Eq. (9) and Eq. (10).

(9) $$\frac{\partial P_{i}}{\partial \delta_{j}}=-\left|V_{i}\right|\left|V_{j}\right|\left|Y_{ij}\right|\text{sin}\left(\theta_{ij}-\delta_{i}+\delta_{j}\right)$$

(10) $$\frac{\partial P_{i}}{\partial \delta_{j}}=\underset{j\neq i}{\sum }\left|V_{i}\right|\left|V_{j}\right|\left|Y_{ij}\right|\text{cos}\left(\theta_{ij}-\delta_{i}+\delta_{j}\right)$$

### 2.2 Power World Simulator

Power world simulator (PWS) software is deployed in this research work for simulation of load flow study; it is a model-based program. Power world simulator is user-friendly and interactive simulation software. The simulator makes use of full-colored animated one-line diagrams. It also possesses zooming and panning attributes that enhance the user's understanding. PWS is an effective tool for handling analysis of power flow, and it is also a very robust package that is capable of handling 250,000 buses.

### 2.3 Generated bus identification numbering types

**Figure 1:**
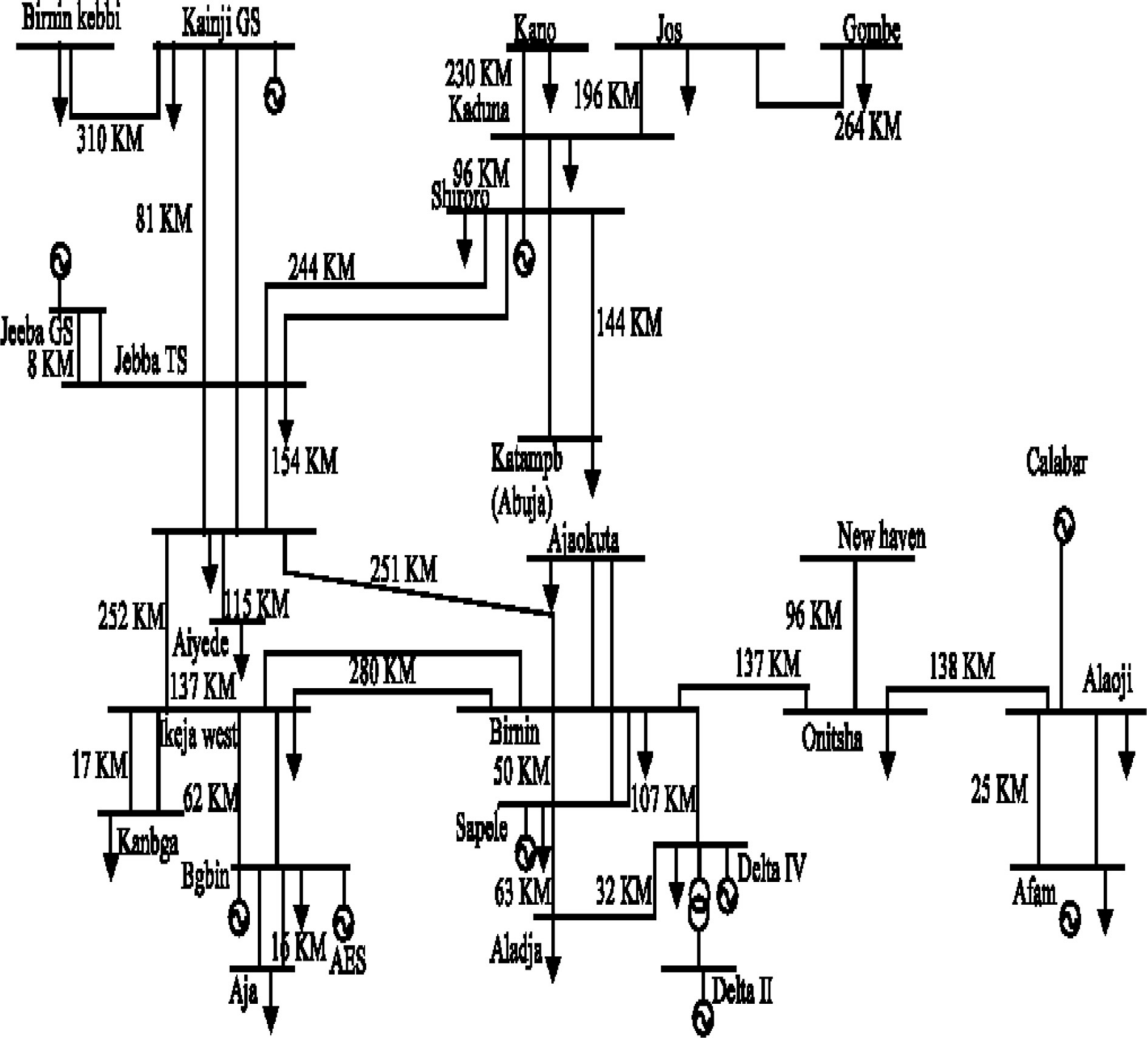
The one-line diagram of the Nigerian existing 28-bus 330-kV power network.

In this study, the Nigerian power network is used as the test system to investigate bus numbering's impact on a radial network. The one-line diagram of the Nigerian existing 28-bus 330-kV power system with the typical bus numbering sequence highlighted in blue is given in Figure 1. The code name and bus numbering that corresponds to this Figure 1 is presented in Table 1.

**Table 1:** The code name and bus numbering that corresponds to Figure 1.

Location	Code	Location	Code	Location	Code	Location	Code
Egbin (1)	EGB	Benin (8)	B	Birnin Kebbi (15)	BK	Kano (22)	KN
Delta IV (2)	DE-IV	Ayede (9)	AY	Gombe (16)	GO	Shiroro (23)	SH
Aja (3)	AJ	Osogbo (10)	OS	Jebba Gs (17)	JG	Sapele (24)	SA
Akangba (4)	AK	Afam (11)	AF	Jebba Ts (18)	JT	Okpai (25)	OK
Ikeja West (5)	IW	Alaoji (12)	ALA	Jos (19)	J	Katampe (26)	KTP
Ajaokuta (6)	AJA	New Heaven (13)	NH	Kaduna (20)	KD	Delta (27)	DE
Aladja (7)	AL	Onitsha (14)	ON	Kainji (21)	KJ	AES (28)	AES

This study presents the bus numbering sequence on the test system that resulted from the generating of the random numbering systems. The bus numbering sequence has impacted load-flow analysis, but must be used in this research. The bus identification numbering case 1 to case 8 is shown in Tables 2, 3, 4, 5, 6, 7, 8, and 9.

**Table 2:** The bus identification numbering case 1.

1	2	3	4	5	6	7	8	9	10	11	12	13	14
EGB	AF	AY	AJ	AJA	AK	AL	ALA	BK	B	DE	GO	IW	JG
15	16	17	18	19	20	21	22	23	24	25	26	27	28
JT	J	KD	KJ	KN	KTP	N-H	OK	DE-IV	ON	OS	AES	SA	SH

**Table 3:** The bus identification numbering case 2.

1	2	3	4	5	6	7	8	9	10	11	12	13	14
KJ	BK	JT	JG	OS	AY	AES	IW	AK	EGB	AJ	DE-IV	B	AJA
15	16	17	18	19	20	21	22	23	24	25	26	27	28
SA	DE	AL	ON	OK	NH	AF	ALA	SH	KTP	KD	KN	J	GO

**Table 4:** The bus identification numbering case 3.

1	2	3	4	5	6	7	8	9	10	11	12	13	14
GO	KJ	JG	KN	KD	AJA	AES	J	AJ	EGB	B	ON	DE-IV	JT
15	16	17	18	19	20	21	22	23	24	25	26	27	28
SA	IW	KTP	DE	OK	AK	ALA	AF	BK	OS	AY	SH	AL	NH

**Table 5:** The bus identification numbering case 4.

1	2	3	4	5	6	7	8	9	10	11	12	13	14
DE	NH	AES	AL	ON	ALA	SA	BK	AF	KN	JT	JG	KD	SH
15	16	17	18	19	20	21	22	23	24	25	26	27	28
AK	B	OK	AJ	J	OS	GO	EGB	IW	AJA	KJ	KTP	DE-IV	AY

**Table 6:** The bus identification numbering case 5.

1	2	3	4	5	6	7	8	9	10	11	12	13	14
BK	KJ	JG	JT	OS	AY	AES	IK	AK	EGB	AJ	KTP	DE-IV	B
15	16	17	18	19	20	21	22	23	24	25	26	27	28
SA	AJA	DE	AL	ON	OK	NH	AF	ALA	SH	KN	KD	J	GO

**Table 7:** The bus identification numbering case 6.

1	2	3	4	5	6	7	8	9	10	11	12	13	14
OS	B	AJ	AJA	IK	NH	AY	J	AF	ON	AK	GO	KTP	EGB
15	16	17	18	19	20	21	22	23	24	25	26	27	28
SH	DE	KD	KN	AL	SA	JG	JT	KJ	ALA	OK	BK	AES	DE-IV

**Table 8:** The bus identification numbering case 7.

1	2	3	4	5	6	7	8	9	10	11	12	13	14
EGB	DE	OK	SA	AF	JG	KJ	SH	AES	DE-IV	OS	B	IW	AY
15	16	17	18	19	20	21	22	23	24	25	26	27	28
J	ON	AK	GO	KTP	AL	KN	AJ	AJA	NH	ALA	JT	BK	KD

**Table 9:** The bus identification numbering case 8.

1	2	3	4	5	6	7	8	9	10	11	12	13	14
AJ	IW	EGB	DE	AK	AJA	AL	B	AY	OS	AF	ALA	NH	ON
15	16	17	18	19	20	21	22	23	24	25	26	27	28
BK	GO	JT	JG	J	KD	KJ	KN	SH	SA	AES	DE-IV	OK	KTP

## 3 Results and discussion

### 3.1 Load flow analysis for the generated bus identification numbering systems using Power World Simulator

**Figure 2:**
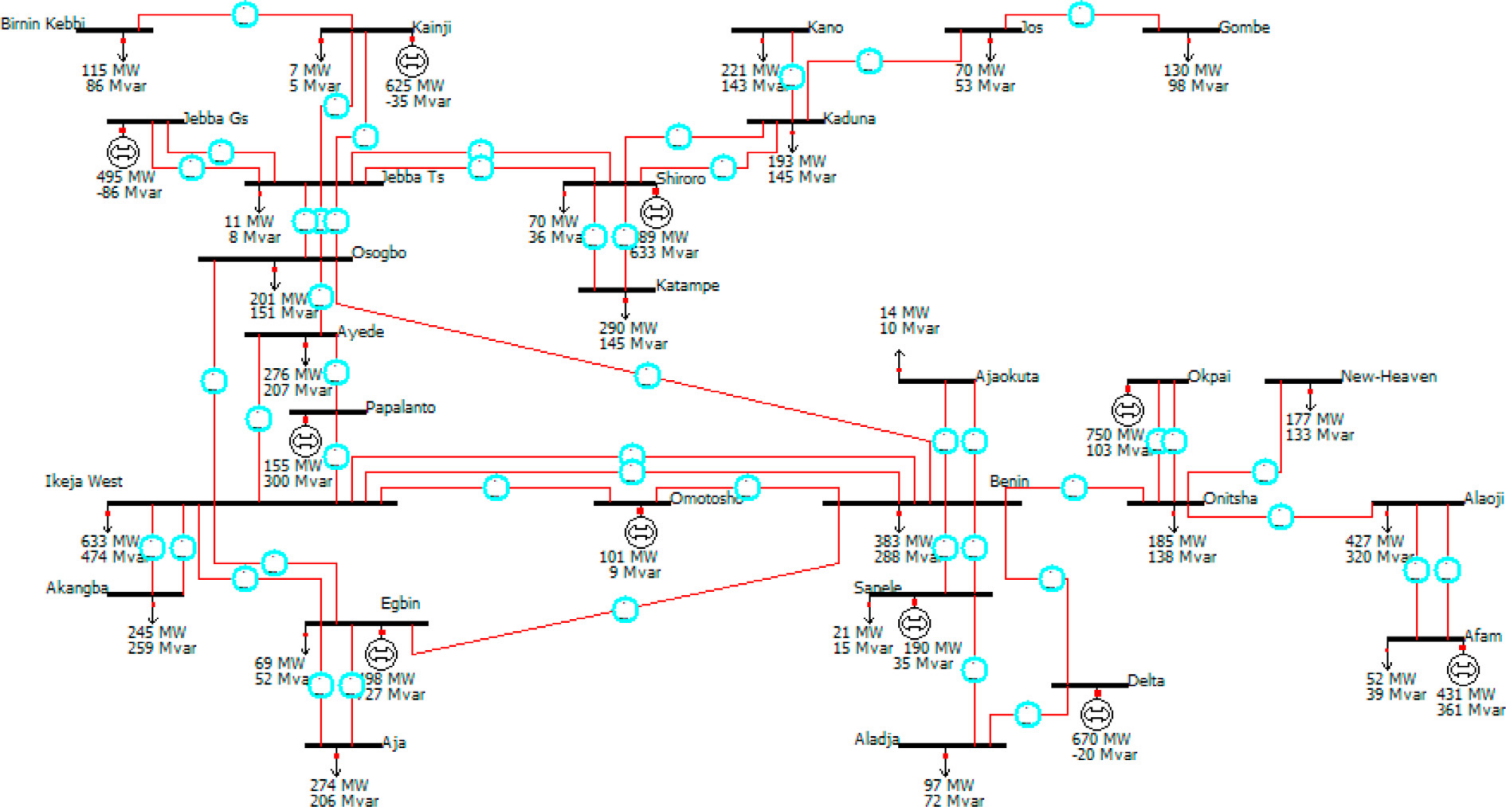
The single-line diagram of the existing 330-kV grid in edit mode of the PWS.

**Figure 3:**
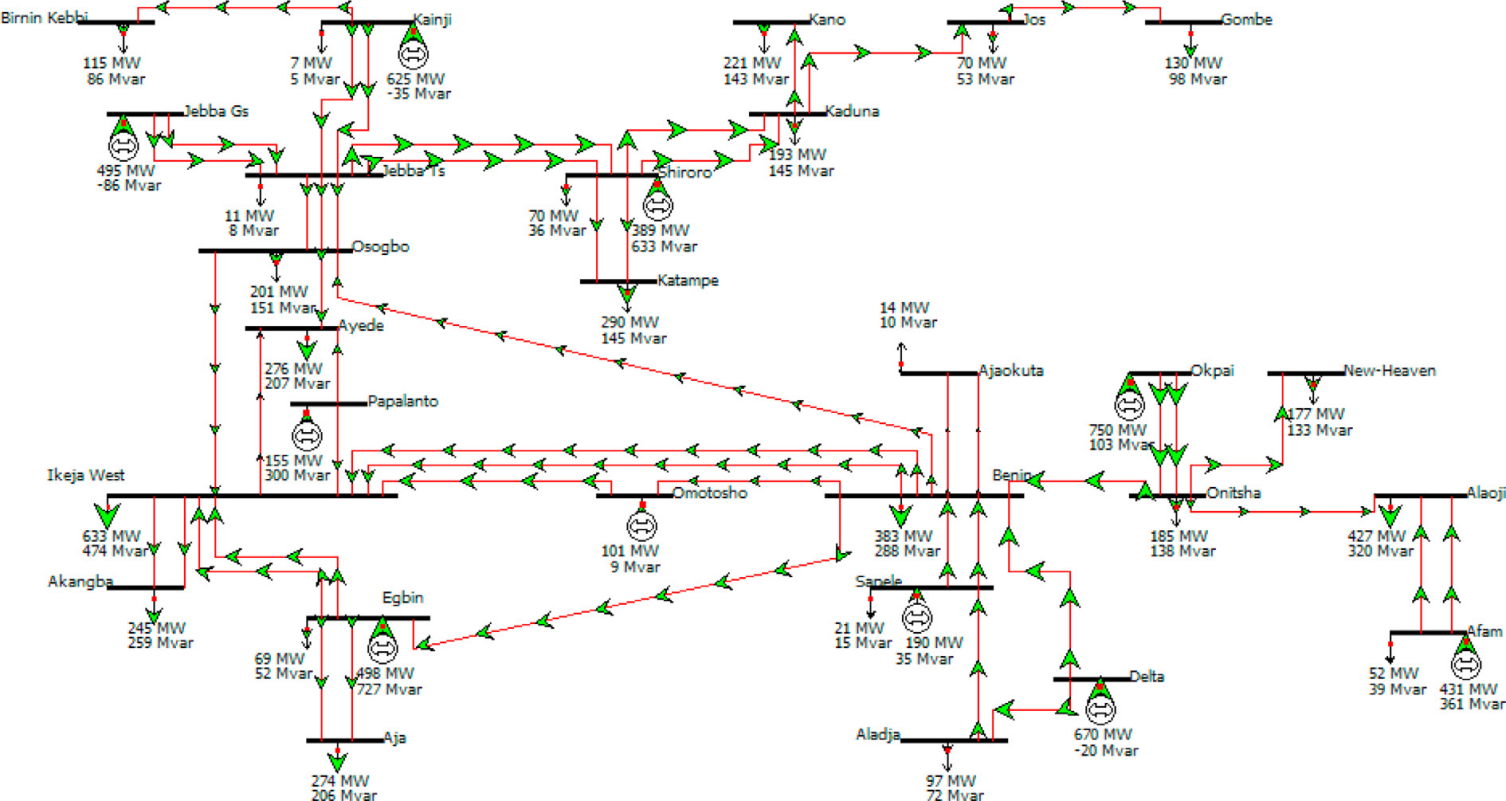
The single-line diagram of the existing 330-kV grid in a run mode of the PWS.

This study's focus is based on the effect of numbering systems generated on the losses, both the real and reactive power losses of the system. The PWS models of the test system both for edit mode and run mode are given in Figures 2 and 3. The losses based on the results from the Power World simulator platform are shown in Tables 10, 11, 12, 13, 14, 15, 16, and 17.

**Table 10:** The line-to-line breakdown of the power losses in case 1.

From	4	10	1	2	3	3	3	5	6	7	7	8	9	10	10	10	10	10
To	1	1	13	8	13	25	26	10	13	11	27	24	18	11	13	23	24	25
MW	0.16	11.5	1.21	5.19	0.13	2.68	3.44	0.02	0.21	1.26	1.41	0.25	2.08	4.27	1.52	2.33	5.43	0.54
Mvar	−2.1	−13	−18	−7.1	−26	−36	−42	−42	−5.2	2.89	−3.1	−26	−46	8.23	−112	−37	12.1	−49.46

From	10	12	13	13	13	14	14	15	15	15	16	17	17	20	21	22	Total Losses	
To	27	16	23	25	26	15	15	18	25	28	17	19	28	28	24	24		
MW	0.69	6.21	8.24	0.33	1.06	0.17	0.17	1.67	0.7	6.11	7.6	8.81	5.79	1.13	1.9	11.8	142.82	
Mvar	−18	13.2	−29	−46	−2.2	−2.3	−2.3	−20	−59	−38	26.6	31.5	12.8	−52	−3.7	−1.9	−1055.5

**Table 11:** The line-to-line breakdown of the power losses in case 2.

From	2	3	4	3	3	6	8	13	6	6	8	9	10	8	13	11	13
To	1	1	3	5	23	5	5	5	7	8	7	8	8	12	8	10	10
MW	2.08	1.66	0.17	0.72	6.42	2.7	0.34	0.54	3.53	0.13	1.1	0.21	1.22	7.99	1.54	0.16	12.59
Mvar	−46	−20	−2.3	−59	−30	−36	−46	−49	−42	−26	−1.9	−5.2	−18	−29	−112	−2.1	−11.21

From	13	14	13	13	13	17	17	19	20	22	21	24	25	25	27	28	Total Losses
To	12	13	15	16	18	15	16	18	18	18	22	23	23	26	25	27	
MW	3.1	0.02	0.71	5.73	5.31	2.85	1.73	11.8	1.9	0.25	5.2	1.27	7.23	11.3	9.59	7.57	157.74
Mvar	−22	−42	−17	20.2	11.3	8.08	6.84	−1.9	−3.7	−26	−7.1	−45	29.2	56.8	46.8	31.9	−880.4

**Table 12:** The line-to-line breakdown of the power losses in case 3.

From	1	14	23	3	5	8	5	6	16	25	9	11	10	11	11	11	11	11
To	8	2	2	14	4	5	26	11	7	7	10	10	16	12	13	15	15	16
MW	6.2	1.67	2.08	0.17	8.81	7.6	5.79	0.02	1.06	3.45	0.16	11.6	1.21	5.42	2.41	0.69	0.69	1.52
Mvar	13.1	−20	−46	−2.3	31.5	26.5	12.8	−42	−2.2	−42	−2.1	−12	−18	12	−37	3.03	3.03	−112.3

From	11	11	19	21	28	16	14	14	27	20	16	25	17	27	22	25	Total Losses	
To	18	24	12	12	12	13	24	26	15	16	24	16	26	18	21	24		
MW	4.26	0.54	11.8	0.25	1.9	8.22	0.71	6.11	1.41	0.21	0.33	0.13	1.13	1.26	5.19	2.68	143.02	
Mvar	8.2	−49	−1.9	−26	−3.7	−29	−59	−38	−3.1	−5.2	−46	−26	−52	2.89	−7.1	−36	−1013.86

**Table 13:** The line-to-line breakdown of the power losses in case 4.

From	4	16	2	23	28	4	6	16	17	9	16	8	13	12	11	11	11
To	1	1	5	3	3	7	5	5	5	6	7	25	10	11	14	20	25
MW	1.26	4.27	1.9	1.06	3.44	1.42	0.25	5.43	11.8	5.19	0.69	2.08	8.81	0.17	6.11	0.7	1.67
Mvar	2.91	8.23	−3.7	−2.2	−42	10.6	−26	12.1	−1.9	−7.1	−18	−46	31.5	−2.3	−38	−59	−19.9

From	13	19	26	15	16	16	16	24	16	18	21	23	28	22	23	28	Total Losses
To	14	13	14	23	20	22	23	16	27	22	19	20	20	23	27	23	
MW	5.79	7.6	1.13	0.21	0.54	11.5	1.52	0.02	2.33	0.16	6.2	0.33	2.68	1.21	8.24	0.13	142.82
Mvar	12.8	26.5	−52	−5.2	−49	−13	−112	−42	−37	−2.1	13.1	−46	−36	−18	−29	−26	−1042

**Table 14:** The line-to-line breakdown of the power losses in case 5.

From	1	4	3	4	4	6	8	14	6	6	8	9	10	8	14	11	14
To	2	2	4	5	24	5	5	5	7	8	7	8	8	13	8	10	10
MW	2.08	1.67	0.17	0.71	6.1	2.69	0.33	0.54	3.44	0.13	1.06	0.21	1.2	8.24	1.52	0.16	11.52
Mvar	−46	−20	−2.3	−59	−38	−36	−46	−49	−42	−26	−2.2	−5.2	−18	−29	−112	−2.06	−12.61

From	12	14	14	16	14	14	18	18	20	21	23	22	26	26	27	28	Total Losses
To	24	13	15	14	17	19	15	17	19	19	19	23	24	25	26	27	
MW	1.13	2.33	0.69	0.02	4.27	5.43	1.41	1.26	11.8	1.9	0.25	5.19	5.76	8.8	7.52	6.12	142.59
Mvar	−52	−37	−18	−42	8.23	12.1	−3.1	2.89	−1.9	−3.7	−26	−7.1	12.5	31.4	25.9	12.35	−1058

**Table 15:** The line-to-line breakdown of the power losses in case 6.

From	2	5	7	22	4	2	2	2	2	2	2	3	7	11	14	5	5
To	1	1	1	1	2	5	10	14	16	20	28	14	5	5	5	27	28
MW	0.54	0.33	2.69	0.71	0.02	1.52	5.43	11.5	4.27	0.69	2.33	0.16	0.13	0.21	1.2	1.06	8.24
Mvar	−49	−46	−36	−59	−42	−112	12.1	−13	8.23	−18	−37	−2.1	−26	−5.2	−18	−2.18	−29.06

From	6	7	12	8	9	24	25	13	17	22	19	17	19	21	22	26	Total Losses
To	10	27	8	17	24	10	10	15	15	15	16	18	20	22	23	23	
MW	1.9	3.44	6.06	7.45	5.19	0.25	11.8	1.13	5.74	6.09	1.26	8.79	1.41	0.17	1.67	2.08	142.41
Mvar	−3.7	−42	11.8	25.3	−7.1	−25.9	−1.9	−52	12.4	−38	2.89	31.3	−3.1	−2.3	−20	−46.38	−1059

**Table 16:** The line-to-line breakdown of the power losses in case 7.

From	12	1	22	12	20	3	12	20	5	6	26	27	19	26	28	13	14
To	1	13	1	2	2	16	4	4	25	26	7	7	8	8	8	9	9
MW	11.5	1.2	0.16	4.27	1.26	11.75	0.69	1.41	5.19	0.17	1.67	2.08	1.13	6.09	5.73	1.06	3.44
Mvar	−13	−18	−2.1	8.23	2.89	−1.94	−18	−3.1	−7.1	−2.3	−20	−46	−52	−38	12.2	−2.18	−41.91

From	12	13	12	13	14	26	12	12	23	14	17	18	15	24	25	28	Total Losses
To	10	10	11	11	11	11	13	16	12	13	13	15	28	16	16	21	
MW	2.33	8.24	0.53	0.33	2.69	0.71	1.52	5.43	0.02	0.13	0.21	6.01	7.4	1.9	0.25	8.79	142.28
Mvar	−37	−29	−50	−46	−36	−58.9	−112	12.1	−42	−26	−5.2	11.2	24.9	−3.7	−26	31.26	−1061

**Table 17:** The line-to-line breakdown of the power losses in case 8.

From	1	3	5	8	9	2	2	2	8	7	8	6	7	8	8	8	8
To	3	2	2	2	2	10	25	26	3	4	4	8	24	10	14	24	26
MW	0.16	1.2	0.21	1.52	0.13	0.33	1.06	8.24	11.5	1.26	4.27	0.02	1.41	0.54	5.43	0.69	2.33
Mvar	−2.1	−18	−5.2	−112	−26	−46.2	−2.2	−29	−13	2.89	8.23	−42	−3.1	−49	12.1	−17.57	−37.39

From	9	9	17	11	12	13	27	15	16	18	17	17	19	20	20	28	Total Losses
To	10	25	10	12	14	14	14	21	19	17	21	23	20	22	23	23	
MW	2.69	3.44	0.71	5.19	0.25	1.9	11.8	2.08	6.12	0.17	1.67	6.1	7.52	8.8	5.76	1.13	142.59
Mvar	−36	−42	−59	−7.1	−26	−3.74	−1.9	−46	12.4	−2.3	−20	−38	25.9	31.4	12.5	−51.94	−1058

**Figure 4:**
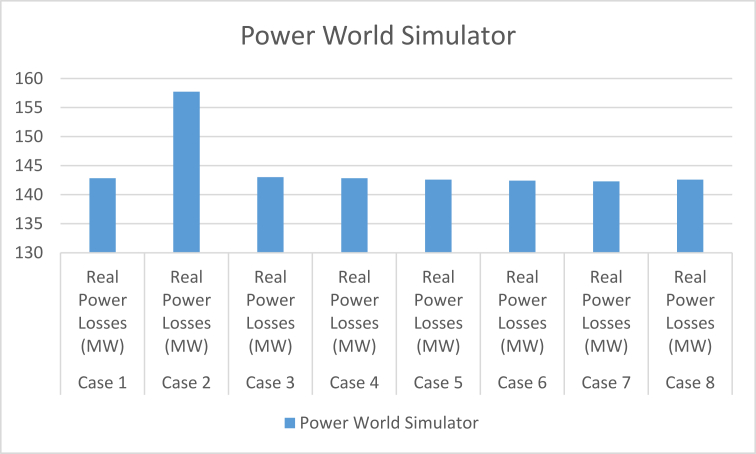
The chart comparing the real power losses between the different identification numbering systems.

**Figure 5:**
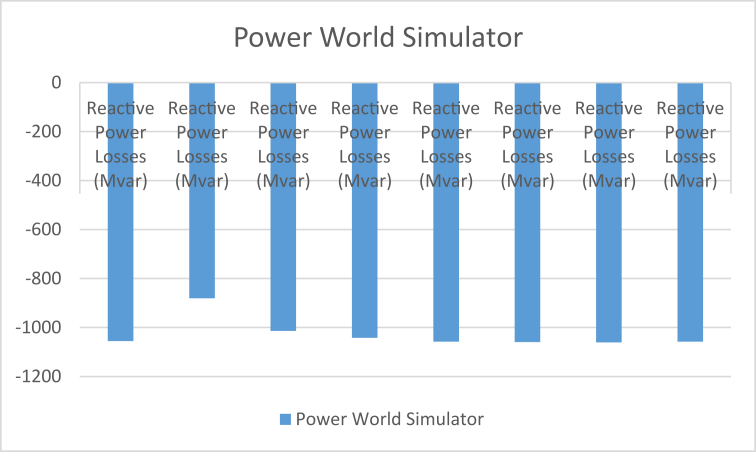
The chart comparing the reactive power losses between the different identification numbering systems.

From the results of Table 11 and Figures 4 and 5, the real and reactive power losses in case 2 are higher than the remaining case studies, but the other results for real are relatively the same but with slight variations to their values. The presence of the relatively large variation in case 2 shows that this study carried out over a larger range of cases will help eliminate the possibilities of using bus identification numbering systems that will contribute to excessive real power losses in our power system. The reactive power losses for all the cases examined in this study have shown that the bus identification numbering system also affects the magnitude of these losses by reducing them or increasing them. From these findings, it can be concluded that the numbering system affects reactive power because of the radial nature of the Nigerian 330-kV power network. The reactive power helps prevent the voltage collapse in the power system and maintain voltage stability of the system. Reactive power is used to provide the voltage levels necessary for real power to do useful work. Reactive power is essential to move real power through the transmission and distribution system to the customer. Hence the need to consider the bus identification numbering system when setting up a power system network.

### 3.2 Load flow analysis using MATLAB

The use of one method or process of carrying out power flowload-flow analysis is not sufficient in the assessment of a power system hence the need for alternate platforms to ensure all parameters are critically examined and confirmed. Therefore, in this study, the MATLAB platform is used to confirm the PWS platform's findings. Unlike the PWS platform, MATLAB is a code-based platform that is considered to be a bit more reliable than the model-based system used in the PWS.

The results using MATLAB are displayed in Table 18 for case 2 and Table 19 for case 4. Case 2 did not converge after 100 iterations. A similar analysis using MATLAB is performed for cases 1, 3, 5, 6, 7, and 8. Cases 4 and 8 converged at the 10^th^ iteration while cases 1, 3, 5, 6 and 7 converged at the 5^th^ iteration. The total active and reactive power losses are respectively **516.48MW + 2,488.086MVAr** for case 2 and **208.89MW - 1,770.879MVAr** for case 4. The case 2 result reveals a considerable high losses for both real and reactive power while case 4 on the other hand, has a reasonable amount of active and reactive power losses but with 10 iteration number to converge compared to others as shown in Table 20.

**Table 18:** The load-flow result for case 2 using the MATLAB platform.

Bus No.	Voltage Mag.	Angle Degree	Load		Generation		Injected
			MW	MVAr	MW	MVAr	MVAr
1	1.050	−3.710	7.000	5.200	624.700	−73.085	0.000
2	1.009	−9.398	114.500	85.900	0.000	0.000	0.000
3	1.045	−10.181	11.000	8.200	0.000	0.000	0.000
4	1.050	−9.643	0.000	0.000	495.000	120.688	0.000
5	1.022	−8.386	201.200	150.900	0.000	0.000	0.000
6	0.999	−8.527	275.800	206.800	0.000	0.000	0.000
7	1.050	−6.271	0.000	0.000	154.800	339.664	0.000
8	1.008	−6.003	633.200	474.000	0.000	0.000	0.000
9	0.994	−6.602	244.700	258.500	0.000	0.000	0.000
10	1.050	0.000	68.900	51.700	871.680	482.806	0.000
11	1.040	−0.570	274.400	205.800	0.000	0.000	0.000
12	1.050	−1.686	0.000	0.000	100.600	28.151	0.000
13	1.044	1.029	383.300	287.500	0.000	0.000	0.000
14	1.061	0.487	13.800	10.300	0.000	0.000	0.000
15	1.050	3.730	20.600	15.400	190.300	28.011	0.000
16	1.050	7.158	0.000	0.000	670.000	−32.528	0.000
17	1.046	5.768	96.500	72.400	0.000	0.000	0.000
18	0.985	7.645	184.600	138.400	0.000	0.000	0.000
19	1.050	10.407	0.000	0.000	750.000	16.015	0.000
20	0.941	4.800	177.000	133.400	0.000	0.000	0.000
21	1.050	5.598	52.500	39.400	431.000	362.503	0.000
22	0.995	5.798	427.000	320.200	0.000	0.000	0.000
23	1.050	−50.693	70.300	36.100	388.900 4	298.860	0.000
24	0.993	−56.844	290.100	145.000	0.000	0.000	0.000
25	0.006	1352.445	193.000	144.700	0.000	0.000	0.000
26	0.204	−2740.959	220.600	142.900	0.000	0.000	0.000
27	0.336	2318.241	70.300	52.700	0.000	0.000	0.000
28	0.243	1947.539	130.200	97.600	0.000	0.000	0.000
**Total**			4160.500	3083.000	4676.980	5571.086	0.000

**Table 19:** The load-flow result for case 4 using the MATLAB platform.

Bus No.	Voltage Mag.	Angle Degree	Load		Generation		Injected
			MW	MVAr	MW	MVAr	MVAr
1	1.050	9.720	0.000	0.000	670.000	−5.799	0.000
2	0.940	7.378	177.000	133.400	0.000	0.000	0.000
3	1.050	−3.409	0.000	0.000	154.800	451.645	0.000
4	1.046	8.327	96.500	72.400	0.000	0.000	0.000
5	0.984	10.228	184.600	138.400	0.000	0.000	0.000
6	0.995	8.370	427.000	320.200	0.000	0.000	0.000
7	1.050	6.285	20.600	15.400	190.300	89.133	0.000
8	1.009	8.548	114.500	85.900	0.000	0.000	0.000
9	1.050	8.160	52.500	39.400	431.000	364.822	0.000
10	0.985	−33.899	220.600	142.900	0.000	0.000	0.000
11	1.049	7.764	11.000	8.200	0.000	0.000	0.000
12	1.050	8.330	0.000	0.000	495.000	−1.826	0.000
13	1.055	−25.752	193.000	144.700	0.000	0.000	0.000
14	1.050	−16.011	70.300	36.100	388.900	115.842	0.000
15	0.986	−4.048	244.700	258.500	0.000	0.000	0.000
16	1.037	3.611	383.300	287.500	0.000	0.000	0.000
17	1.050	12.927	0.000	0.000	750.000	31.357	0.000
18	1.040	−0.570	274.400	205.800	0.000	0.000	0.000
19	1.141	−32.396	70.300	52.700	0.000	0.000	0.000
20	1.022	−0.790	201.200	150.900	0.000	0.000	0.000
21	1.152	−37.152	130.200	97.600	0.000	0.000	0.000
22	1.050	0.000	68.900	51.700	564.091	681.050	0.000
23	1.001	−3.440	633.200	474.000	0.000	0.000	0.000
24	1.054	3.064	13.800	10.300	0.000	0.000	0.000
25	1.050	14.235	7.000	5.200	624.700	−83.981	0.000
26	0.993	−22.162	290.100	145.000	0.000	0.000	0.000
27	1.000	2.919	0.000	0.000	100.600	−330.122	0.000
28	0.999	−3.902	275.800	206.800	0.000	0.000	0.000
**Total**			4,160.500	3,083.000	4,369.391	1,312.121	0.000

**Table 20:** The power losses comparison between the PWS and the MATLAB.

Case Type	Loss Type	Power World Simulator	MATLAB	No of Iterations Using MATLAB
Case 1	Real Power Losses (MW)	142.82	200.998	5
	Reactive Power Losses (Mvar)	−1055.5	−1736.529	
Case 2	Real Power Losses (MW)	157.74	516.48	101 (Not converge)
	Reactive Power Losses (Mvar)	−880.38	2488.086	
Case 3	Real Power Losses (MW)	143.02	196.283	5
	Reactive Power Losses (Mvar)	−1013.86	−1822.734	
Case 4	Real Power Losses (MW)	142.82	208.891	10
	Reactive Power Losses (Mvar)	−1042.04	−1770.879	
Case 5	Real Power Losses (MW)	142.59	200.984	5
	Reactive Power Losses (Mvar)	−1057.83	−1760.17	
Case 6	Real Power Losses (MW)	142.41	196.283	5
	Reactive Power Losses (Mvar)	−1059.47	−1822.734	
Case 7	Real Power Losses (MW)	142.28	196.232	5
	Reactive Power Losses (Mvar)	−1060.89	−1825.124	
Case 8	Real Power Losses (MW)	142.59	195.482	10
	Reactive Power Losses (Mvar)	−1057.84	−1813.477	

### 3.3 Verification of power losses using MATLAB

**Figure 6:**
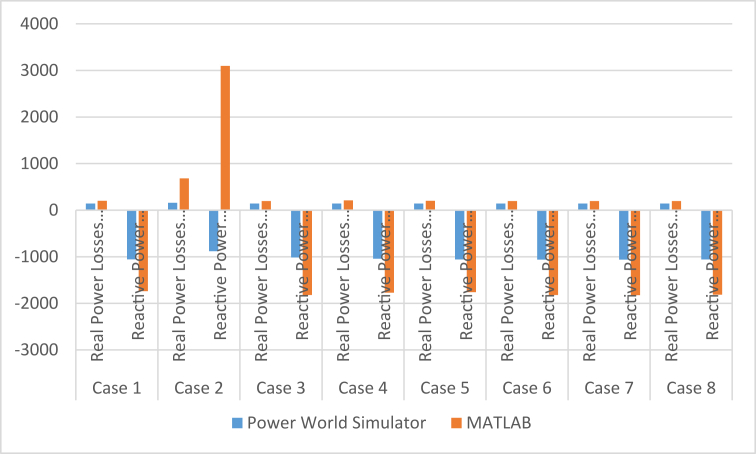
A chart comparing the real and reactive power losses between using the PWS and the MATLAB.

The results of Table 20 show a direct comparison between the power losses for both real and reactive between the PWS Platform, which is a model-based platform and the MATLAB platform which is a code-based platform. There are slight variations to the results, as shown in Figure 6. This is due to their different approaches to carrying out load-flow using the Newton Raphson iterative method, hence the need to use more than one platform for the analysis. The variations in the real and reactive power losses, as well as the variation in the number of iterations in solving for each case while using the MATLAB platform also helps to strengthen the conclusion of this study that the bus identification numbering system affects the flow of power both real and reactive in a power system network.

## 4 Conclusions and recommendations

The results presented in this study show that bus identification numbering type affects the power system's losses. The study discovered that the bus numbering sequence in case 2 failed to converge and aborted at iteration number 101. The consequence is that this in-convergent bus identification model type is difficult to solve on the test network (radial) using a single software or single iterative method due to insufficient reactive power flow. The results obtained from the PWS and the MATLAB platform presented complement each other.

The result presented in the load-flow result for case 2 using the MATLAB platform (Table 18) shows that case 2 has the lowest convergence rate and the highest power losses on both the Power World Simulator and the MATLAB platform. Therefore, it is not advisable to use the bus numbering arrangement of case 2. Further, that case 7 in Table 20 shows that case 7 has a comparable convergence rate based on the iterations carried out to complete its load flow analysis. The power losses on both the MATLAB and Power World Simulator given in section 3.1 and section 3.3 are the lowest amongst all the cases considered. Therefore, it can be established that case 7 presents the most suitable bus identification numbering type among all the cases considered, hence recommended for use on this test system for load-flow study for a more reliable result.

For increased credibility of results, more load-flow analysis methods like the Fast-Decoupled as well as the recently developed Holomorphic embedding should be used in further studies. Also, other code-based platforms like MATLAB should be used to replace model-based platforms like the Power World Simulator Platform for increased accuracy.

## Declarations

### Author contribution statement

Ademola Abdulkareem: Conceived and designed the experiments; Performed the experiments; Contributed reagents, materials, analysis tools or data; Wrote the paper.

Alayande A. S.: Contributed reagents, materials, analysis tools or data; Wrote the paper.

Somefun T. E.: Performed the experiments; Analyzed and interpreted the data; Wrote the paper.

Ette E. V.: Conceived and designed the experiments; Performed the experiments; Analyzed and interpreted the data; Contributed reagents, materials, analysis tools or data; Wrote the paper.

### Funding statement

This work was supported by Covenant University Centre for Research, Innovation and Discovery.

### Data availability statement

Data included in article/supp. material/referenced in article.

### Declaration of interests statement

The authors declare no conflict of interest.

### Additional information

No additional information is available for this paper.
